# On the Removal of Stains in Linen Made by Nitrate of Silver

**Published:** 1847-05

**Authors:** W. B. Herapath

**Affiliations:** Lond., Bristol


*On the removal of Stains in Linen made by Nitrate of Silver.* By W. B. Herapath, M. B. Lond., Bristol.—Medical practitioners in the habit of using the nitrate of silver extensively, as a remedial agent, must have frequently heard loud complaints of their patients' linen having been indelibly stained and spoilt, by some accident having occurred during its use ; and in many cases, patients have refused to employ these preparations, in consequence of the extensive destruction of linen which they occasion. I have therefore very little doubt that the following observations will prove most acceptable to my brother practitioners :—

These dark stains consist of very finely divided metallic silver in intimate union with the fibres of the cloth. Had they been oxide of silver, any diluted acid would have dissolved them ; but nitric acid alone produces any effect upon them, which of course cannot be employed on account of its powerfully destructive effects upon the linen fabric. Iodine immediately converts them into iodide of silver, which is instantly dissolved by a solution of hypo-sulphate of soda, and the cloth remains as white as when issued from the bleaching house, and as firm and durable as ever.

The best mode of employing the substance is to strain the spotted linen over a basin of hot water, and then to let fall upon each spot, previously moistened with water, a few drops of tincture of iodine, and instantly to pour a sufficient solution of the hypo-sulphate of soda to dissolve the iodide produced, and then immerse the spot in the water beneath, to wash out and cleanse the tissue, at once, from the stain and chemical reagents employed. The tincture of iodine of London Pharmacopœia strength is the one I employ ; and one drachm of crystallized hypo-sulphate of soda, dissolved in two ounces of water, will make an excellent bleaching liquid.

A patient may thus be very readily taught the manner of removing an unpleasantness frequently attending the use of a most valuable remedy.—*London Lancet.*

